# The role of flaps and grafts in modern hypospadiology

**DOI:** 10.4103/0970-1591.40616

**Published:** 2008

**Authors:** M. Chad Wallis, Luis Braga, Antoine Khoury

**Affiliations:** Department of Surgery, Division of Urology, University of Utah and Primary Children’s Medical Center, Salt Lake City, UT 84113, USA; 1 Department of Surgery, Division of Urology, University of Toronto and The Hospital for Sick Children, Toronto, ON, Canada

**Keywords:** Free grafts, hypospadias, outcomes, preputial flaps, urethroplasty

## Abstract

The modern hypospadiologist must be proficient in the use of both vascularized flaps and free grafts. When choosing a repair for any given patient with hypospadias, one must consider the length of the urethroplasty, the presence and degree of ventral curvature and perhaps most importantly, the surgeon’s own experience. Not all repairs are created equally and different complication rates and cosmetic outcomes can be seen among different surgeons utilizing the same technique. Each surgeon tends to infuse their own modifications to any given technique and many of these modifications go unreported. It is incumbent upon each surgeon to be familiar with a wide variety of techniques, which invariably includes the use of flaps and grafts. We present a spectrum of the uses of flaps and grafts in modern hypospadiology.

## INTRODUCTION

Numerous procedures and techniques have been described to correct hypospadias. The use of vascularized skin flaps and free grafts to repair hypospadias began in the late 19^th^ century. In 1861, Bouisson was the first to describe the use of a flap to repair hypospadias. The technique utilized a parameatal based scrotal flap that is strikingly similar to the Mathieu procedure. Novι-Josserand is credited with the first successful use of a free graft to create a neourethra in 1897.[1] Tubularization of the urethral plate for repair of hypospadias was described by Thiersch in 1869 and Duplay in 1874.[2] In truth, all techniques reported since the late 1800s have been variations on these early themes.

Few would argue that Snodgrass’ modification of the Thiersch-Duplay technique, the tubularized incised plate (TIP) urethroplasty,[3] has gained widespread acceptance all over the world. Cook *et al.* reported a recent survey of 101 physicians who regularly perform hypospadias surgery. Those surveyed included physicians from the Americas and Europe. The results indicate that there is a strong preference for utilizing the TIP technique, particularly in cases of distal and midshaft hypospadias without chordee.[4] While this may have had the effect of diminishing the use of flaps and grafts in hypospadias surgery, it has by no means eliminated their use. The experienced reconstructive surgeon is able to recognize those cases where the deficiency in the ventral components of the penis (skin, urethral plate, and corpora) are significant enough to justify addition of new tissue in the way of a graft or a flap. Thus, they remain an important element of surgical training for physicians that are intent on making hypospadias surgery a component of their practice.

Flaps are transferred from tissues in the vicinity of the penis using vascularized pedicles, while free grafts are being harvested from more remote locations on the body [Table 1]. In addition, the clinical uses for flaps and grafts in hypospadias surgery have expanded over time [Table 2]. However, the principles established more than 100 years ago remain the same today. Proper dissection and tissue handling are critical components to the use of flaps and grafts.

**Table 1:** Commonly used flaps and grafts described in hypospadias surgery

Vascularized flap	Free graft
Parameatal Preputial Tunica vaginalis Scrotal	Preputial skin Bladder mucosa Buccal mucosa Posterior auricular skin

**Table 2:** Common uses flaps and grafts in hypospadias surgery

Vascularized flap	Free graft
Urethral augmentation Substitution urethroplasty Waterprofing layer Skin resurfacing Ventral lengthening for correction of ventral curvaturee	Urethral augmentation Urethral substitution Skin resurfacing Ventral lengthening for correction of ventral curvatur

## USES OF VASCULARIZED FLAPS

### Urethral augmentation

As previously described, the use of flaps for hypospadias repair began in the late 19^th^ century with Bouisson’s description of a parameatal flap to perform an onlay urethroplasty. Urethral augmentation using an onlay technique is preferred by many over urethral substitution because it allows for preservation of the natural urethral plate and does not rely on a vascular pedicle or neovascularization of a free graft for the entire circumference of the urethra. A variety of techniques have been described using parameatal flaps to augment the urethral plate in creating a neourethra. The best known of these for use in distal hypospadias is likely the Mathieu technique, initially described in 1932.[5] The basic technique involves elevating a flap of skin on the ventral shaft of the penis proximal to the hypospadiac meatus that is roughly the same length as the distance from the meatus to the tip of the penis. The flap should be designed such that the length is not more than double the width. The proximal free end of the flap is then “flipped” distally and the flap is anastamosed to the urethral plate as an onlay urethroplasty. Other techniques such as the Mustarde and the “flip-flap” as described by Horton and Devine are essentially modifications of the Mathieu, though the Horton-Devine technique uses a tubularized parameatal flap instead of an onlay.[6,7] A major criticism of all these techniques has been the abnormal appearance of the meatus. Both the hinge technique, described by Rich and the MAVIS modification were designed to correct for this and produce a more natural appearing glans and slit-like meatus.[8,9]

Several large series have reported using the Mathieu technique or one of its modifications. A series by Retik *et al.* included 204 patients with follow-up ranging from 6 months to 6 years and complication rate of 0.98%.[10] Another report consisted of a multicenter retrospective review of patients undergoing a modified Mathieu procedure by four different surgeons and included 336 patients.[11] The overall complication rate was 3.3% with fistula being the most common complication. The study compared patients who were stented postoperatively vs. those who were not stented and found no statistically significant differences in complication rates. Minevich *et al.* reported their series of 202 patients who underwent a Mathieu hypospadias repair.^12^ They had the longest follow-up ranging from 25 to 83 months and a complication rate of 1.5% with fistula formation in two patients and meatal retraction in one. The technique offers low complication rates in these large studies; however, cosmetic appearance continues to be a concern when compared to the more popular TIP repair.[13,14]

The use of inner preputial skin on a vascularized pedicle for urethroplasty was first described by Hook in 1896.[5] Use of such a flap for augmentation of the native urethral plate, known as an onlay island flap (OIF), did not come into common use until Elder *et al.* reported the technique in 1987.^15^ Although initially described as a technique for midshaft and distal hypospadias, over time it has been used increasingly for more proximal repairs, while the TIP repair is now used more commonly in midshaft and distal repairs.[4] Therefore, most series have consisted of patients with more proximal hypospadias and this has resulted in much smaller series to report and higher complication rates.

Ghali published a series of 418 primary single-stage hypospadias repairs with a mean follow-up of 23 months.[16] The series included 42 patients who underwent an OIF repair. This included 12 patients with distal and 30 patients with midshaft/proximal hypospadias. The overall complication rate using the OIF technique was 3%. In 1997, Wiener *et al.* reported their series of patients with proximal hypospadias who underwent repair utilizing a preputial island flap. Their series included 58 patients who were repaired using an onlay technique and their overall complication rate was 31% with a mean follow-up of 20 months.[17] Recently, Sedberry-Ross *et al.* reported on their 17-year experience with split prepuce *in situ* onlay hypospadias repair involving 421 patients with all types of hypospadias. The overall complication rate was 22.5% and the average time between operation and complication presentation was 19 months, suggesting that small fistulas become clinically apparent only after toilet training.[18] Finally, our recent series from Toronto compared the OIF to TIP repair at a single center for penoscrotal hypospadias.[19] We reported on 40 patients who underwent OIF with an overall complication rate of 45% with a mean follow-up of 38 months. Although OIF procedure was associated with a significantly lower fistula rate when compared to TIP repair (20% vs. 43%), recurrent ventral curvature was more frequent after OIF.

### Substitution urethroplasty

Proximal hypospadias is more frequently associated with severe ventral curvature. There are recent trends toward preservation of the urethral plate at all costs; however, there remain some patients who require transection of the urethral plate at the time of primary repair. The threshold at which an individual surgeon is apt to proceed with transection of the urethral plate is obviously variable. Once the threshold has been crossed, the question left to answer is whether to perform a one-stage or two-stage urethroplasty. Those who favor one-stage repairs, generally select a tubularized flap to perform a substitution urethroplasty once the ventral curvature has been corrected. In addition, a tubularized flap for urethral substitution may be used in cases where an existing urethral plate is deemed “unsuitable” for an onlay or TIP technique such as in reoperative cases.

The preputial tubularized island flap is perhaps the most popular option among hypospadiologists. Though others throughout the world helped to pioneer the technique,[20,21] it is Duckett who typically receives credit for making the technique a popular choice for proximal hypospadias.[22]

Ghali’s previously mentioned series included 148 patients who underwent repair using the Duckett technique. All patients were described as having a midshaft or proximal hypospadias with moderate or severe ventral curvature. An overall complication rate of 32% was reported.[16] Wiener *et al.* also reported the results of 74 patients who underwent a Duckett procedure with a 36% complication rate. The series included patients with distal and proximal hypospadias as well as varying degrees of ventral curvature. Perhaps the largest series of tubularized flaps is the one reported by Liem *et al.*[23] The technique used was a modification of the original Duckett transverse island flap. In this series of 176 patients, a longitudinal island flap was harvested using preputial and penile skin. Patients were described as having penile or more proximal hypospadias. The authors reported a 7.4% fistula rate that was, not surprisingly, higher in patients with more severe hypospadias.

The Koyanagi-Nonomura one stage repair utilizes a meatal-based preputial flap for substitution urethroplasty as a one stage repair for severe hypospadias.[24] This technically challenging technique has been shown to have a higher complication rate, up to 46% in a series of 70 patients reported by Koyanagi *et al.*[25] The technique was modified by Emir *et al.* to improve the vascularity of the flap, resulting in more acceptable complication rates.[26]

### Intermediate layer for fistula prevention

Fistula formation has long plagued those who perform hypospadias surgery. The low fistula rates in series of Mathieu repairs are thought to be a result of the fact that there are no overlapping suture lines in a Mathieu repair as there are in the Thiersch-Duplay or TIP repair. Smith was the first to describe using a de-epithelialized skin flap as an intermediate layer between the neourethra and overlying skin.[27] De-epithelialized preputial and parameatal flaps have been described resulting in fistula rates of < 5%.[10,28,29] Other techniques have been described using tunica vaginalis, scrotal dartos, and external spermatic fascia flaps with decreased fistula rates.[30–32] A recent prospective randomized study evaluated patients who had a TIP repair with and without a subcutaneous ventral flap. They randomized 130 patients and found a statistically significant reduction in the number of fistulas among patients who had a flap as an intermediate layer.[33] Due to the improvements in fistula rates, creating some form of vascularized flap as an intermediate barrier is now common practice in hypospadias surgery.

### Skin resurfacing

Ventral skin deficiency is frequently seen in patients with hypospadias. Skin coverage of the penile shaft at the end of a hypospadias repair can be challenging and the skin deficiency may become more prominent following correction of ventral curvature and/or the use of ventral skin flaps during urethroplasty. Byars described a multistage repair for hypospadias in 1951 using flaps created from the dorsal preputial skin.[5] The skin is incised longitudinally and then translocated to the ventral aspect of the penile shaft for use in creating a neourethra at a subsequent stage. These same Byar’s flaps have come into routine use for resurfacing the ventral penile shaft in most hypospadias repairs.

Preputial transverse island flaps have also been used to resurface ventral skin defects.[34,35] This has been taken a step further with the use of double face transverse island flaps being utilized both to perform the urethroplasty and to cover a ventral skin defect.[36–38] The technique has been reported with good results, though redundancy of the ventral skin particularly along the sides is a cosmetic issue.

### Correction of ventral curvature using flaps

A variety of alternative techniques are available for repair of severe chordee[39–41]; however, for most, it comes down to two main philosophies. One philosophy is to perform dorsal plications, which may result in shortening of the penis. The other philosophy is that of lengthening the penis by incising the corporal bodies and placing a patch over the defect. Free grafts have been reported more frequently in the literature; however, we have previously reported animal studies that demonstrated improved viability with the use of a vascularized tunica vaginalis flap when compared to free grafts.[42] We also recently reported our experience with tunica vaginalis flap for ventral lengthening in 38 boys with severe ventral curvature.[43] A free graft (dermal or lyophilized bovine dura) was combined with a tunica vaginalis flap in 11 patients, 23 were repaired with a tunica vaginalis flap alone and the remaining four underwent ventral corporal grafting alone due to associated bilateral cryptorchidism. With a median follow-up of 5.3 years, recurrence of chordee occurred in five patients. Of the 23 patients who were repaired with a tunica vaginalis flap alone; only one patient developed recurrence, whereas four of nine patients who were grafted with dura developed recurrence. Although initial results are encouraging, longer follow-up is required in this series of patients.

## FREE GRAFTS

Free grafts, when used in any kind of reconstruction, bear the distinct disadvantage of requiring neovascularization to occur in order to sustain healthy, viable tissue. Having said that, an advantage to using a free graft is that graft material is more easily obtainable, particularly in reoperative cases. More frequently, grafts have been used in cases where there is poor blood supply to local tissues due to previous surgeries or deficiency of local skin as in severe cases of hypospadias where a staged approach may be preferred. As a result, series tend to be smaller, complication rates higher and primary repairs are frequently combined with redo hypospadias repairs in many series.

### Urethral augmentation

A variety of tissues have been used as free grafts for the urethroplasty of patients with hypospadias, though buccal mucosa is becoming the favored graft material for this application today. Buccal mucosal is thought to have optimal vascular characteristics due to the presence of a panlaminar plexus, which allows thinning of buccal mucosal grafts while preserving the physical characteristics. Because a free graft relies on neovascularization, one would intuitively assume that an onlay or inlay technique would result in healthier tissue than a tubularized substitution urethroplasty. A recent meta-analysis of the use of oral mucosa grafts in hypospadias/epispadias reconstruction confirms this assumption. The authors reported 362 cases where an onlay graft was used with a success rate of 80.4% vs. a 52.7% success rate among tube grafts (55 cases).[44]

In cases where the native urethral plate is deemed inadequate, a dorsal inlay graft may be performed to augment the urethral plate, which may then be tubularized in a Thiersch-Duplay fashion. Both preputial skin and buccal mucosa have been successfully used.^45^

### Substitution urethroplasty

Free grafts were first used for urethroplasty by Novι-Josserand in 1897. He used a split thickness free graft and tubularized it around a metal probe to create a neourethra.[1] Devine and Horton described the use of a full thickness preputial graft that was tubularized in a single stage repair.[6] Use of skin grafts in urethroplasty, both split thickness and full thickness, have been hampered by hair growth, graft contraction, and BXO. In 1947, Memmelar was the first to describe the use of bladder mucosa in a one-stage repair.[5] Others have tried bladder mucosa with some success; however, reports of meatal complications have made this a less desirable material in the distal urethra. A recent series from China included 294 patients who underwent a composite tubularized bladder mucosa-skin graft with an overall complication rate of only 12.3%.[46] These authors have overcome previous issues of meatal prolapse and stenosis by using a tubularized skin flap for the distal urethra. As indicated previously, efforts at using buccal mucosa as a tubularized free graft proved to have lower success rates when compare to onlay techniques.[44]

Perhaps the greatest use of free grafts for hypospadias repair comes into use with a staged procedure. The technique is most frequently used for proximal hypospadias with severe chordee or in reoperative cases where there is limited local skin and blood supply is compromised. Popularized recently by Bracka, the technique has proved reliable in a variety of settings.[47] Inner preputial skin, when available and buccal mucosa are the most popular choice of material in this setting.

Finally, advances in the field of tissue engineering have already begun to show promise for creating suitable tissues, grown *in vitro*, which may be used as a urethral substitute. Several animal models have already been reported[48,49] and we await the report of clinical trials using this technology.

### Skin resurfacing

Although resurfacing of the penile shaft is most frequently performed using local skin flaps, those patients who have undergone multiple repairs may have a deficiency of local skin for such use. In instances where penile skin is deficient, both split thickness and full thickness skin grafts have been applied for skin coverage.[50,51] Principles of skin transfer must be adhered to in order to ensure adequate take of the graft. Scar tissue must be excised and a vascular bed prepared for the graft.

### Correction of ventral curvature using grafts

When a ventral lengthening procedure is chosen for correction of severe ventral curvature, free grafts have been reported more commonly than vascularized flaps. A number of materials have been utilized including dermal grafts, tunica vaginalis free grafts, dura, pericardium, and small intestinal submucosa (SIS).[43] As indicated previously, our animal studies suggested that a vascularized flap may have an advantage over free grafts and our clinical experience supported this. Subsequent animal studies, however, have suggested that SIS may be comparable to a tunica vaginalis flap.[52,53] Indeed, SIS has been reported with increasing frequency in recent years as a material of choice in ventral lengthening procedures. However, laying down a free graft on the ventral surface of the penis might result in a three-stage repair if a free graft also needs to be used for the urethroplasty as it is not possible to lay a graft on top of a graft.

## CONCLUSION

Individual hypospadiologists must use the techniques that have the greatest success rates in their own hands. While recent trends have lead to a decreased emphasis on flaps and grafts for creating a neourethra in more distal hypospadias, flaps remain a popular choice for an intermediate layer to prevent fistula in these cases. In more proximal or reoperative cases, when additional tissue is required for reconstruction of the urethra, ventral lengthening of the corpora or for skin cover, surgeons must have at their disposal a broad knowledge of the uses of flaps and grafts to ensure the best outcomes for their patients.
